# Non-target effects on soil microbial parameters of the synthetic pesticide carbendazim with the biopesticides cantharidin and norcantharidin

**DOI:** 10.1038/s41598-017-05923-8

**Published:** 2017-07-17

**Authors:** Hainan Shao, Yalin Zhang

**Affiliations:** Key Laboratory of Plant Protection Resources and Pest Management, Ministry of Education, College of Plant Protection, Northwest A&F University, Yangling, Shaanxi 712100 China

## Abstract

Considering the fact that biopesticides are increasingly used to replace synthetic pesticides in pest control, it is necessary to assess their ecotoxicity and especially their non-target effects on soil microorganisms, which is largely unknown. In this study, the effects of the synthetic pesticide carbendazim and the biopesticides (cantharidin and norcantharidin) on soil microbial parameters in a silt loam soil were evaluated. By using commercial formulations at the recommended and higher rates, both cantharidin and norcantharidin induced adverse effects on soil invertase, phosphatase activities and fungal gene structure, but these changes were transient. After about two weeks, the harmful effects owing to the application of pesticides phased out and eventually became comparable with non-treated samples. The degradation of cantharidin and norcantharidin was rapid and can be completed within a few days in the soil. None of the three pesticides caused significant shifts in urease activity. This study provides a comprehensive assessment of the soil microbial toxicity of these biopesticides for reasonable and efficient usage.

## Introduction

Over the past 100 years, population growth has forced the improvement of global agricultural production^1^. However, agriculture is always adversely affected by pests (insects, weeds and plant pathogens) which cause up to 45% of crops to be lost annually^2^. Application of pesticides is therefore a common and critical method to improve crop yield^3–5^. Of the tremendous amount of pesticides used, more than 99.7% persist and are accumulated in the environment^6–8^, undergoing various physicochemical catabolism and biodegradation processes which are closely associated with the composition and activity of soil microbial community^9–11^. Soil microorganisms have key functions in many vital soil processes, such as organic matter decomposition and nutrient cycling, and are responsible to a great extent for the function of soil ecosystems^12^. Hence, any loss of soil microbial community structure could result in significant shifts in soil fertility which is pre-requisite for plant growth.

An ideal pesticide should not adversely affect organisms other than its targeted pests. Due to the natural origin (from natural plants, animal toxins and living microbes), biopesticides are considered to be safer or to have evolved to have low non-target toxicity. By using a variety of detection tests (polyphasic microbial assays, DGGE and soil microbial biomass, etc.), recent studies suggested that biopesticides can exhibit high but transient biocidal effects. Sameh *et al*. (2007) found that paenimyxin produced by *Paenibacillus* sp. strain B2 had a negative effect on the soil bacterial community^13^. Hernández *et al*.^14^ showed that the community structure of ammonia-oxidizing bacteria was altered by the herbicide simazine^14^. Muñoz-Leoz *et al*.^15^ demonstrated that the ethofumesate herbicide, deltamethrin insecticide and difenoconazole fungicide produced significantly adverse impacts on soil enzymes and overall soil microbial activity^15^. Singh *et al*.^16^ reported that cypermethrin and a low dose of azadirachtin showed a negative impact on the resident and active rhizospheric bacterial community^16^. Nevertheless, to our knowledge, these studies generally focused on the influence of chemosynthetic pesticides and biopesticides of plant or microbial origin, while no data are available with regard to the impact of animal-derived pesticides on such community guilds.

Carbendazim is a systemic benzimidazole fungicide used to control a broad range of diseases on arable crops (e.g., *Fusarium graminearum* and *Rhizoctonia solani*)^17, 18^, fruits (e.g., *Botryosphaeria berengeriana* and *glomerella cingulate*)^19^, vegetables (e.g., *Alternaria solani* and *Sclerotinia sclerotiorum*)^20^, ornamentals and medicinal herbs^21^. Previous studies have reported that carbendazim had an inhibitory effect on soil parameters such as soil respiration, enzymatic activities and soil fungal: bacterial ratios and could impede other fungicides’ dissipation in the soil^22^. It is of importance to note that repetitive and extensive applications of carbendazim have resulted in the emergence and prevalence of carbendazim resistance in most plant pathogens^18, 20^. Due to the deleterious effects of chemical pesticides, biopesticides, with a new mode of action, higher effectiveness and eco-friendliness should be individually-, sequentially- and simultaneously-applied to maintain crop productions. As two animal-derived compounds, cantharidin and norcantharidin exhibit a similar action mode: they both can inhibit protein serine/threonine phosphatases (PSPs) *in vivo* ^23^. Protein phosphorylation and dephosphorylation act on living cells to participate in a suite of cellular metabolic activities in response to changing circumstances, internal developmental cues or external environmental stimuli in eukaryotes^24^. Even though cantharidin and norcantharidin have been utilized as pesticides, limited work has been done to study the effect of cantharidin and norcantharidin on pest control and anti-pathogenic fungi^25, 26^.

Carbendazim has been extensively used in Chinese agriculture and displays a similar *in vitro* antifungal spectrum to cantharidin and norcantharidin against plant pathogenic fungi to some extent, so it was chosen for the present study. Carbendazim was compared with cantharidin and norcantharidin to assess the disturbance they cause in the soil ecosystem. Considering the ubiquity and the conserved catalytic domain of PSPs in eukaryotes, it is necessary to evaluate the impact of cantharidin and norcantharidin on soil fungal community and in-depth analysis of carbendazim by qualitative analysis should also be attempted.

Assessing the toxicity of biopesticides on soil microbial communities is a pre-requisite to improve pesticides regulation in the near future. Most studies only consider the culturable fraction of soil microorganisms when evaluating the non-target effects of pesticides. In this study, we offer a comprehensive understanding of the potential ecological risks of diverse pesticides on the soil ecosystem. 18S rRNA PCR-denaturing gradient gel electrophoresis (PCR-DGGE) and the enzymatic activities of important nutrient cycles are used in combination to evaluate the effects of cantharidin and its analogue norcantharidin on soil microbial eco-toxicity. The treated-soil quality index (T-SQI) proposed by Mijangos *et al*.^27^ was calculated as well based on the values of the enzymatic activities^27^.

## Results

### Effects of pesticides on microbial parameters in soil

**Figure 1:**
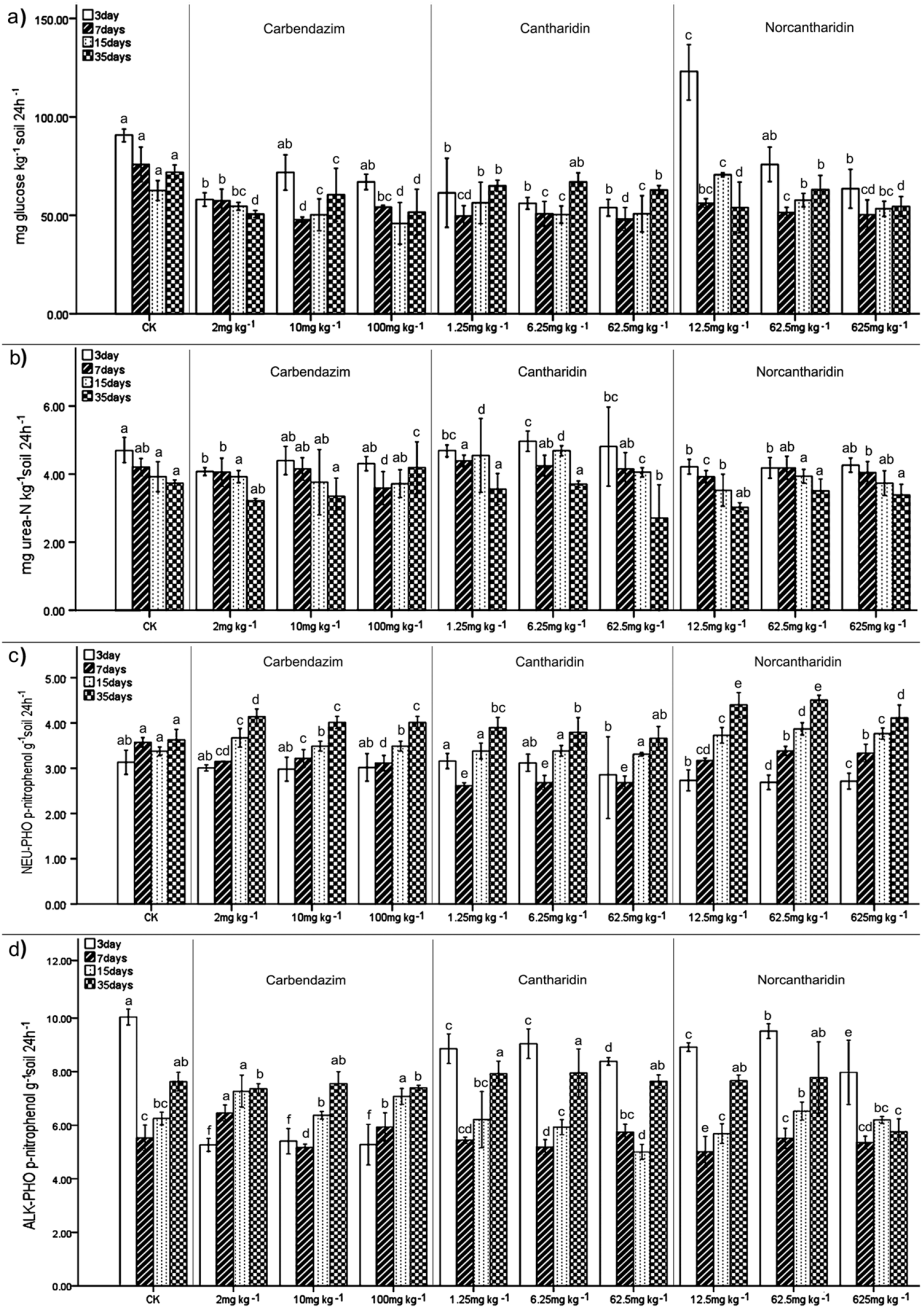
Effect of pesticides (cantharidin, norcantharidin, carbendazim) at recommended rate, five-fold and fifty-fold, on soil enzymatic activities. Mean values (n = 3) ± S.D. (**a**) Invertase activity, (**b**) Urease activity, (**c**) Neutral phosphate activity, (**d**) Alkaline phosphatase activity. Different letters indicate a statistically significant difference at the confidence level of 0.05 between treatments at the same time.

A general reduction in the invertase activity with pesticides application in the soil was evident (Supplementary Table S1, Fig. 1a). According to Fig. 1a, considerably lower values of invertase activity were found on day 7 for both cantharidin- and norcantharidin-treated soils, even upon exposure to the lowest concentrations compared with the untreated soils; similarly, in soils treated with norcantharidin and carbendazim at fifty-fold, significant decreases in invertase activity (on average, 39.4% and 32.0% lower, respectively) were detected at all incubate time compared to the counterparts of control. In addition, on days 15 and 35, the addition of 10 and 100 mg kg^−1^ DW of carbendazim significantly decreased this enzyme activity. By contrast, invertase activity showed higher values on day 3 than on days 7, 15 and 35 for carbendazim- and norcantharidin- treated soils. In norcantharidin-treated soils, significantly higher values of invertase were observed at 12.5 *versus* 62.5 mg kg^−1^ DW on day 3. The detrimental effect because of pesticides application on soil invertase gradually weakened over with time.

Pesticides appear to have no clear effect on soil urease activity at all incubation times at 5 and 50-fold, excluding the addition of 6.25 mg kg^−1^ DW which led to significantly higher values of urease activity in cantharidin-treated samples on day 15 (Fig. 1b); likewise, higher values were generally found in samples treated with RC on days 3, 7 and 15. In carbendazim-treated soil, higher values of urease were observed at 100 *versus* 10 mg kg^−1^ DW on 35, whereas significantly lower values were observed at 100 mg kg^−1^ DW compared with non-treated soils on day 7.

The effects of pesticides on the activities of alkaline phosphate and neutral phosphatase are depicted in Fig. 1c and d. Compared with untreated soils, a noticeable dose-dependent behavior was observed. Various changes of phosphatase activities at all treated soils were consistent with the trend of untreated samples throughout the entire incubation time (Table 1). Neutral phosphatase activity was significantly inhibited (*P* < 0.05) in cantharidin- and norcantharidin-treated soils on days 3 and 7, whereas carbendazim had no clear effect on neutral phosphatase activity. The inhibition effect of cantharidin and norcantharidin increased with the increasing concentrations, as indicated by the stronger effect of the 50-fold treatments in comparison to the RC treatments. However, the stimulatory effects of pesticide-treated samples on neutral phosphate activity were generally found on days 15 and 35, particularly in soils treated with 12.5 and 62.5 mg kg^−1^ DW of norcantharidin (Fig. 1c, Table 1). According to the Fig. 1c, in cantharidin-treated soils, the values of neutral phosphatase activities were the same as the control on day 15, regardless of the concentrations. However, in norcantharidin-treated soils, higher values of neutral phosphatase activities were found on day 15 independent of the concentrations.

**Table 1:** Effect of carbendazim, cantharidin and norcantharidin on the treated–soil quantity (T-SQI) at 3, 7, 15, 35 days of incubation in the presence of different concentrations of pesticides.

pesticide	Pesticide concentration (mg kg^−1^)	Time (days)			
		3	7	15	35
carbendazim	2	23.8 ± 11.0^d^	97.0 ± 8.1^a^	103.3 ± 5.1^a^	63.9 ± 8.9^d^
	10	33.9 ± 11.4^c^	94.3 ± 8.6^ab^	69.8 ± 6.0^c^	71.9 ± 6.3^c^
	100	31.0 ± 12.4^c^	94.7 ± 6.9^ab^	102.2 ± 9.4^a^	75.3 ± 9.7^c^
cantharidin	1.25	52.0 ± 10.7^b^	93.8 ± 10.2^b^	95.9 ± 6.4^a^	88.1 ± 3.5^b^
	6.25	55.4 ± 10.6^b^	93.3 ± 8.5^b^	86.0 ± 7.4^b^	92.2 ± 2.2^b^
	62.5	34.7 ± 9.2^c^	93.4 ± 10.3^b^	60.4 ± 6.4^cd^	21.7 ± 2.9^f^
norcantharidin	12.5	63.0 ± 2.0^a^	94.7 ± 4.4^ab^	94.3 ± 5.9^ab^	66.0 ± 10.3^cd^
	62.5	55.9 ± 2.8^b^	95.8 ± 7.9^ab^	104.2 ± 4.1^a^	192.4 ± .0.7^a^
	625	38.4 ± 5.7^c^	94.7 ± 8.4^ab^	84.2 ± 5.1^b^	53.2 ± 8.5^e^

Mean values (n = 3) ± S.D. values followed by different letters are significantly different at each incubation time according to Fisher’s PLSD test (P < 0.05). Reference value (100%): pesticide-free controls.

Figure 1d shows the variation of alkaline phosphatase after three commercial pesticide applications. Values of alkaline phosphatase were significantly lower on day 7 than on days 3, 15 and 35 in all pesticide-treated soils (Fig. 1d). However, on day 7 soils treated with 2 and 100 mg kg^−1^ DW of carbendazim produced higher values than on day 3 when obviously inhibition of alkaline phosphatase was found even upon exposure to the lowest concentration of carbendazim compared to untreated soils. In addition, the inhibition effect of carbendazim was greater than that of cantharidin and norcantharidin on day 3. Obviously, in cantharidin-treated soils, higher values of alkaline phosphatase were observed at 62.5 *versus* 6.25 and 1.25 mg kg^−1^ DW on day 7. No clear differences were observed between cantharidin- and norcantharidin-treated and control soils, while carbendazim application showed a stimulatory effect on soil alkaline phosphatase in comparison with untreated controls on day 15. On the 35th day, the activity of alkaline phosphatase in pesticide-treated soils was gradually increased to match that of the control, while the alkaline phosphatase activity was still inhibited at 625 mg norcantharidin kg^−1^ DW. Pesticide concentrations had an erratic effect on soil enzymes and an inverse relationship was observed between samples treated with carbendazim, cantharidin and norcantharidin on days 3 and 7 (Supplementary Table 1).

**Figure 2:**
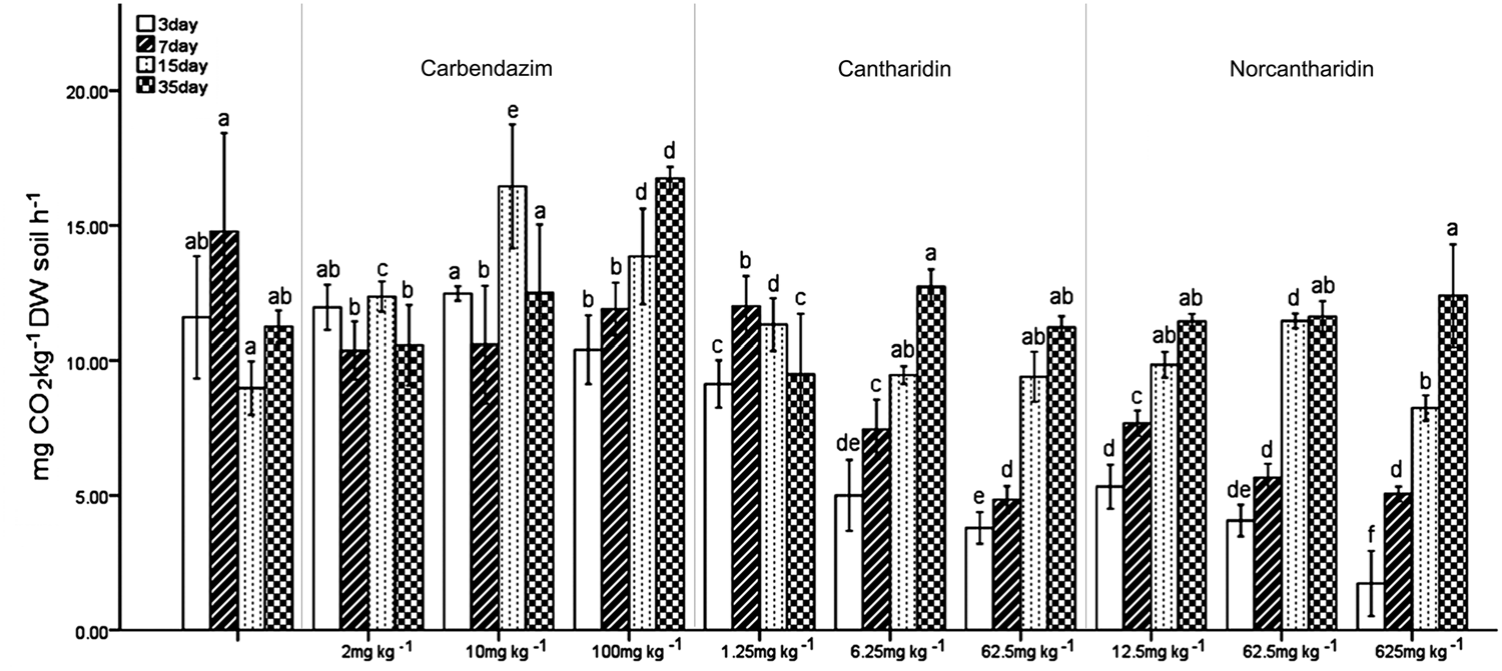
Soil microbial basal respiration of soils treated with carbendazim, cantharidin and norcantharidin during the 35-day incubation (mean ± S.D.). Letters near data points indicate a significant difference between the pesticide treatment and controls.

Regarding soil basal respiration (SIR) (Fig. 2), at 5- and 50-fold recommended rates, conspicuously lower values of SIR were observed on days 3 and 7 for both cantharidin- and norcantharidin-treated soils. Moreover, in carbendazim-treated soils, lower values of SIR were observed at 2 and 10 *versus* 100 mg kg^−1^ on day 35. However, in the case of cantharidin- and norcantharidin-treated soils, significantly higher values of SIR were found on days 15 and 35 *versus* on days 3 and 7, suggesting that the addition of pesticides caused a stimulatory effect on microbial biomass (as measured by SIR) in all samples. Indeed, in norcantharidin-treated samples, higher values of SIR were observed at 62.5 *versus* 12.5 and 625 mg kg^−1^ DW on day 15. By contrast, there is no significant difference (*P* < 0.05) between the samples treated with cantharidin and norcantharidin and the control till the end of the experiments.

The T-SQI can integrate information from different enzyme activities into one unique measure of soil functioning. On day 7, pesticide application showed no clear significant effect on T-SQI values (apart from the cantharidin-treated soils) (Table 1). In contrast, on days 3, 15 and 35, the index exhibited conspicuous changes, especially in samples treated with 62.5 mg kg^−1^ DW of cantharidin and norcantharidin on day 35 showing the lowest and highest values (21.7 ± 2.9 and 192.4 ± 0.7; *p* < 0.05), respectively. Carbendazim induced a distinct decrease of soil quality on day 3 before the detrimental impact gradual recovery on the studied soils. As a general trend, it was found that the higher the pesticide concentration, the lower the T-SQI value.

### Degradation of cantharidin and norcantharidin in soil

For the soil fortified with norcantharidin at three concentrations of 12.5, 62.5 and 625 mg/kg, the recoveries were 87.4%, 86.3% and 91.2% with a relative standard deviation (RSD) of 5.8%, 6.7% and 0.4%, respectively. The recoveries of cantharidin of 1.25, 6.25 and 62.5 mg/kg were 81.72%, 88.56% and 98.73% and with coefficients of variation (CV) of 1.2%, 5.4% and 8.3%. The residual dynamics of cantharidin and norcantharidin in treated soils are shown in Table 2. The evolution of pesticide concentration in soil fits more accurately with the first-order dynamical reaction equation. In cantharidin-treated soil, the half-lives were 0.78, 1.25 and 2.23 days for 1.25, 6.25 and 62.5 mg/kg, respectively. The corresponding values of norcantharidin are 1.32, 1.68 and 1.94 days for 12.5, 62.5 and 625 mg/kg, respectively. The dissipation of these two active ingredients was quite fast and can be almost entirely dissipated in soil after 35 days.

**Table 2:** Degradation dynamics data of cantharidin and norcantharidin at the recommended, five and fifty–fold doses in the soil.

Pesticide treatment	Concentration (mg/kg DW)	Degradation equation	Correlation coefficient	Half-life (d)
Cantharidin	1.25	R = 1.08*e* ^−0.8821t^	0.8144	0.78^a^
	6.25	R = 10.53*e* ^−0.6008t^	0.7212	1.25^b^
	62.5	R = 56.46*e* ^−0.31t^	0.8736	2.23^c^
Norcantharidin	12.5	R = 11.34*e* ^−0.5231t^	0.8654	1.32^a^
	62.5	R = 96.82*e* ^−0.4102t^	0.8231	1.68^ab^
	625	R = 513.27*e* ^−0.3561t^	0.9015	1.94^b^

Data in the same column followed by the different letter are significant (*p* ≤ 0.05).

### Pesticide impact on soil microbial parameters

**Figure 3:**
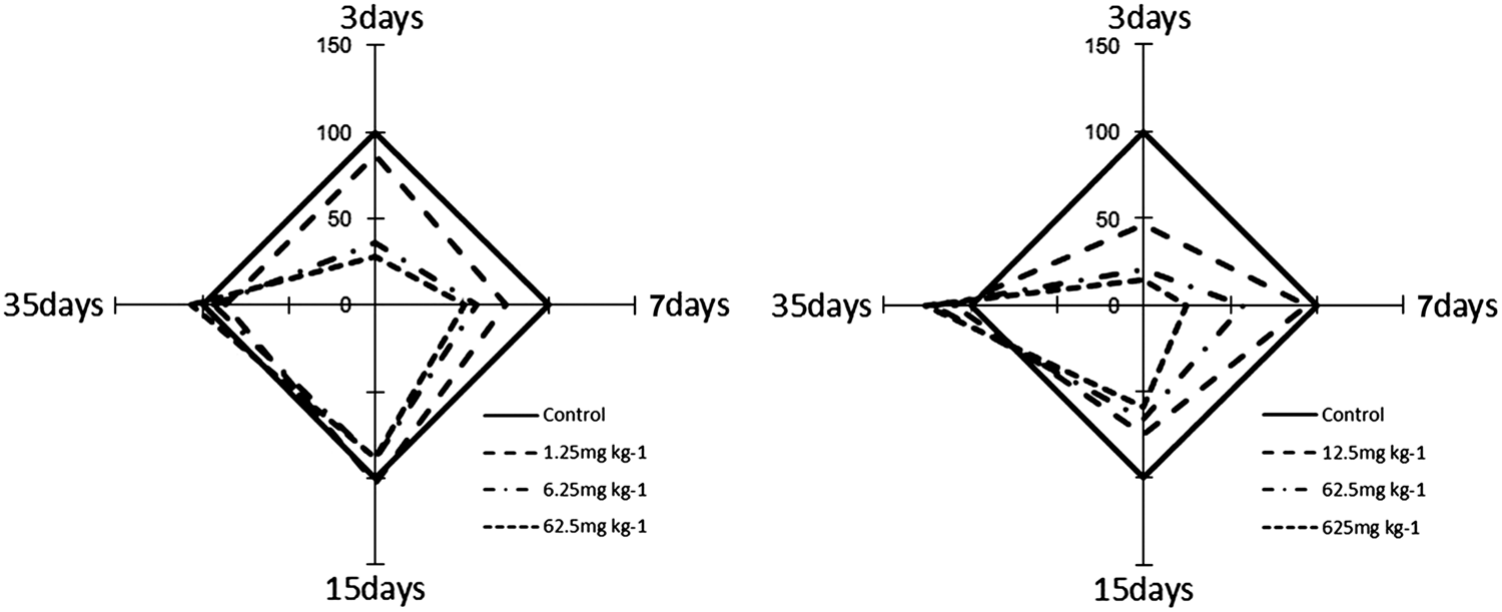
Effect of cantharidin (at 1.25, 6.25 and 62.5 mg kg^−1^ DW soil) and norcantharidin (at 12.5, 62.5 and 625 mg kg^−1^ DW soil) on diversity of fungal population (as reflected by the *H*’ from PCR-DGGE). At each incubation time, values of *H*’ without pesticide treatment was used as reference samples (set to 100%).

**Figure 4:**
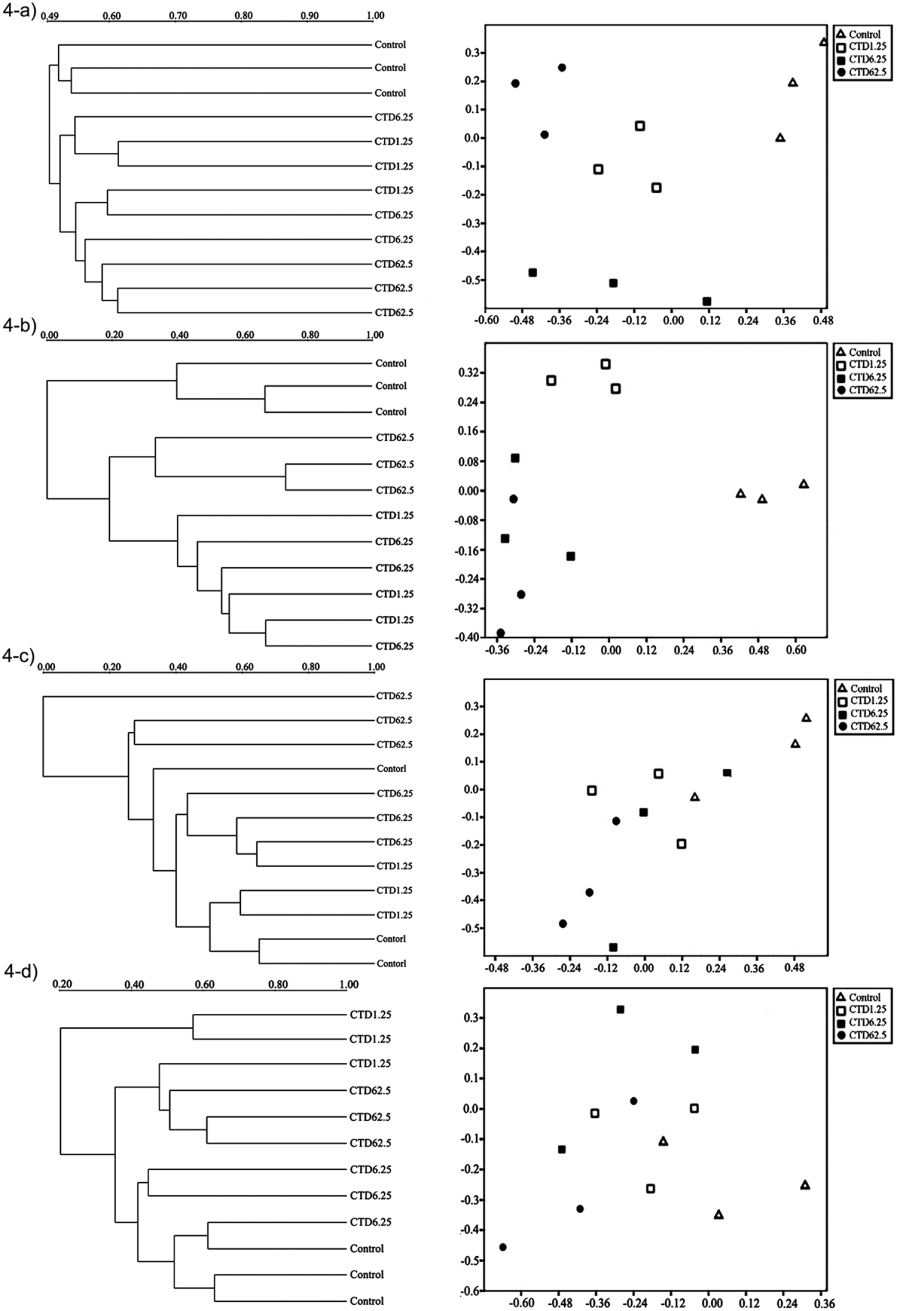
UPGMA and nMDS analyses of 18S rRNA gene from soil fungus. (**a**–**d**) Samples treated with cantharidin (1.25, 6.25 and 62.5 mg kg^−1^ DW soil) and control at different incubation times (**a**, 3 days; **b**, 7 days; **c**, 15 days and **d**, 35 days).

**Figure 5:**
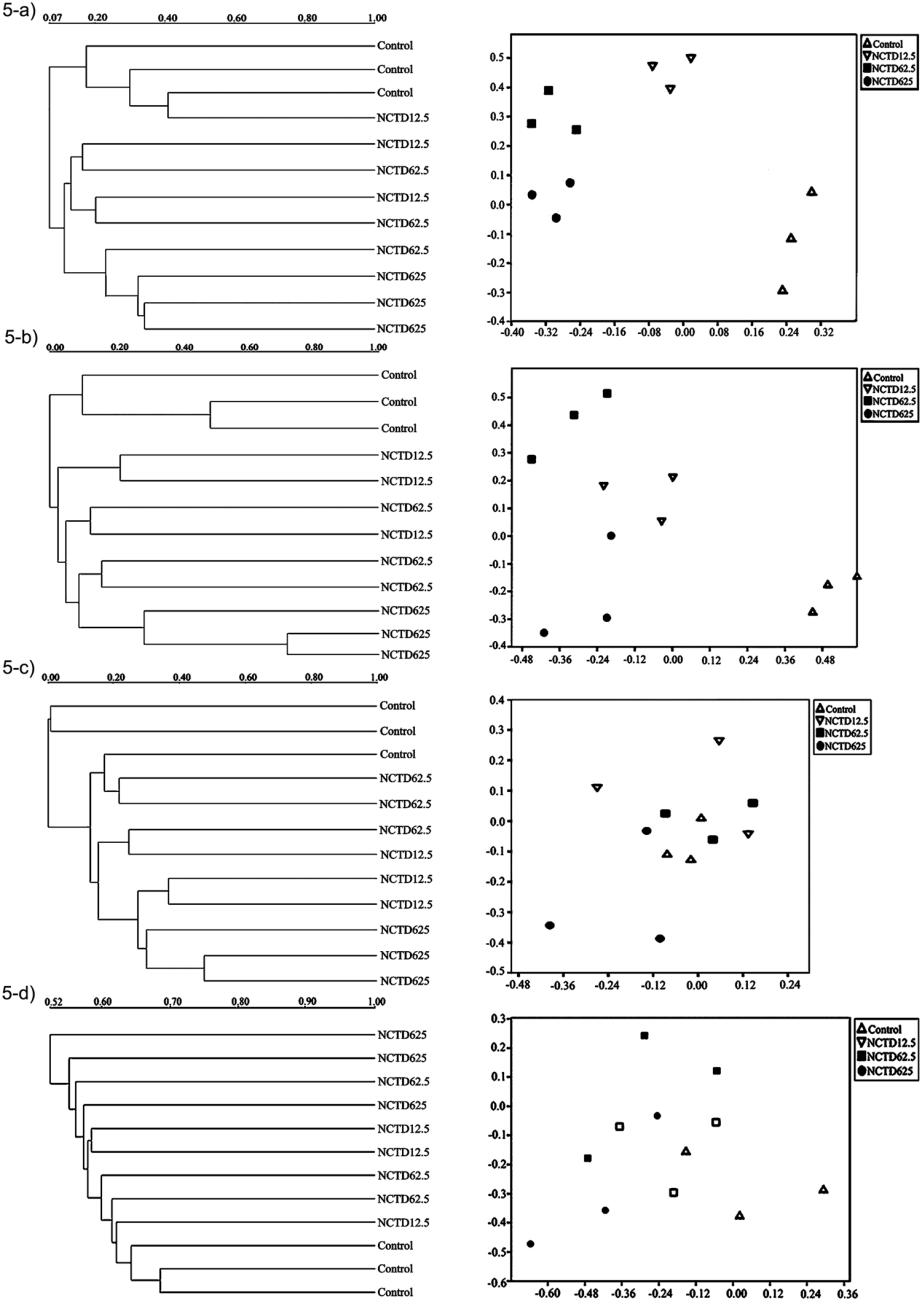
UPGMA and nMDS analyses of 18S rRNA gene from soil fungus. (**a**–**d**) Norcantharidin (12.5, 62.5 and 625 mg kg^−1^ DW soil) treated samples and control at different incubation times (**a**, 3 days; **b**, 7 days; **c**, 15 days and **d**, 35 days).

In our study, microbial parameters were susceptible to the pesticides type and concentration (Supplementary Table S2). Regarding biological pesticides, the effects induced by cantharidin and norcantharidin on the entire soil fungus followed a similar pattern (Fig. 3). In cantharidin- and norcantharidin-treated soils, pesticides induced a marked decrease in fungal population abundance on days 3 and 7, then the inhibitory effect gradually weakened along with the incubation time. The PCR-DGGE fingerprinting with various parameters demonstrated that shifts of microbial communities of the treated and non-treated soils were different because of the usage of pesticides (Figs 4 and 5). Under the application of cantharidin and norcantharidin case scenarios tested, pesticides induced significant alterations at both diversity and functional levels. In particular, cantharidin and norcantharidin substantially decreased the diversity of the fungal community on the 3rd and 7th days compared to untreated soils (Figs 4-a,-b, 5-a and -b). The higher the concentration is, the more obvious the effect is. However, the soil microflora diversity gradually increased after 15 days incubation and was comparable with or even exceeded those values of untreated samples on day 35 (Fig. 4-c and -d). In norcantharidin-treated soils, on days 3 and 7, the diversity of fungal genetic structure clearly declined *versus* that of cantharidin-treated samples (Fig. 3), whereas 62.5 and 625 mg kg^−1^ DW treatments of norcantharidin led to weakly significant changes on day 15 (Fig. 5-c and -d).

## Discussion

In spite of the well-acknowledged role of microflora in soil ecosystem services, the risk assessment of pesticides regarding their soil microbial toxicity remains under-studied, solely relying on simple C and N mineralization tests. In our investigation, short-term side effects of these pesticides tested on soil microbial activity are transient, and the results indicate that the overall influence of pesticides on soil microbiome composition can be attributed to not only the different types of soil properties, but also to the action mode of pesticides.

It is crucial to elucidate the fate of a pesticide for environmental risk assessment, particularly in soils where the microbial communities interact with plants to accomplish functions supporting a number of ecosystem services. Indeed, pesticide dissipation in soil can be attributed to a range of abiotic factors (adsorption, chemical transformation, hydrolysis and photolysis), transfers (leaching and runoff) and biotic processes (adsorption, biological transformation and microbial degradation), not only depending on the physicochemical properties of the pesticide and soil, but also environmental conditions and management practices. In our study, the degradation rates of cantharidin and norcantharidin were quite rapid, possibly due to their anhydride structures which are prone to hydrolyze under alkaline conditions provided by the soil circumstance. Previous studies have demonstrated that isoproturon-degrading bacteria are only isolated in weakly alkaline soils, suggesting that pesticide degradation is strongly linked to soil pH^28, 29^. In addition, soil microorganisms can degrade both parent compounds and corresponding metabolites, but are often more efficient at degrading a metabolite than the parent compound^30^.

The proximate agents of organic matter decomposition are extracellular enzymes (EE). Their production can be viewed as a form of foraging strategy that has evolved whereby nutrient and energy supplies are aligned with demand^31^. Our results show that the application of the synthetic pesticide carbendazim and the two biopesticides cantharidin and norcantharidin induced significant effects on the enzymes in soil (Fig. 1). These soil enzymes play a key role in the transformation of organic matter and also combine with microorganisms to degrade xenobiotics^12, 32^. Invertase is an enzyme involved in the C-cycle process that also facilitates the hydrolysis of organic matter; this is important for increasing the nutrients available in soil. Compared to the untreated controls, the invertase activity values of the three formulated pesticide-treatments were lower, indicating microbial activity was suppressed by cantharidin, norcantharidin and carbendazim (Fig. 1-a). However, both stimulation and inhibition of invertase as a result of pesticide application, has been reported^33–35^.

Urease, as a hydrolase, is involved in the hydrolysis of urea to carbon dioxide and ammonia to be assimilated by soil microbes and plants; it is also a biological indicator in the nitrogen cycle^36^. Extracellular phosphatases are of interest for their role in mineralizing P from nucleic acids, phospholipids and ester phosphates released in the soil. Alkaline phosphatases are exclusively produced by soil microorganisms^37^. Therefore, evaluation of the influence of pesticides on soil extracellular enzymes should include phosphatases and urease because of their important roles participating in metabolism and circulation of key nutrients. Generally, it is thought that phosphatases are insensitive to the toxic effects of pesticides because being an extracellular enzyme, phosphatase is bound with soil colloids and humic substances during degradation. Nevertheless, our results found that the cantharidin and norcantharidin application inhibited the phosphatase activities at an initial stage; then gradually increased until it was comparable with the untreated controls (Fig. 1c and d). The impacts of pesticides (e.g., insecticides, fungicides and herbicides) on phosphatases and urease activities in soils have been studied extensively with contrasting results. Phosphatases are sensitive to such exposure, even at the lowest concentration levels. For example, Yao *et al*.^38^ reported that acetamiprid (chloronicotinyl insecticide) exhibited a significant inhibitory effect on phosphatase activity at 5 mg kg^−1^ DW over the incubation time^38^. During a transient experimental period, napropamide showed a negative effect on acid phosphatase and alkaline phosphatase at field rate^39^. Similarly, detrimental effects on phosphatases were also observed in samples treated with the sulfonylurea herbicide-rimsulfuron and the imidazoline herbicide-imazethapyr^40^. However, stimulatory trends of phosphatase and urease were found when treated with a recommended dose of the pesticide cocktail-Falcon 460 EC fungicide (a mixture of spiroxamine, tebuconazole and triadimenol)^41^ and diazinon even at lower concentrations^42^. On the other hand, Cycoń *et al*.^42^ also found that diazinon application at the recommended rate induced drastically adverse but transient effects on the microbial urease-producing community over the entire sample time^43^.

All soils showed relatively high levels of ureases in our study (Fig. 1-b). It is usually accepted that soil exhibit appreciable urease activity, which is mainly extracellular and particularly persistent because of their association with inorganic and organic colloids. Other studies suggested that a considerable amount of the total activity of an enzyme in soil does not contribute to the measured activity of a soil due to the enzymatic fraction locating either within proliferating and non-proliferating cells or attaching to or containing within cell debris^44^. In addition, the stimulatory effect of phosphatases after 15 days incubation may be ascribed to the adaptive capabilities of soil microorganisms which contribute to minimizing the negative influence of chemical stressors under adverse conditions. Furthermore, the metabolites of pesticides could provide nutrients and energy to a microbial community to accelerate the syntheses of extracellular enzymes.

There is considerable research focusing on the effect of herbicides and insecticides on soil microbial communities^27, 45–48^, compared to fungicides which have been reported to induce more non-harmful effects on soil microbical communities^6, 46, 49, 50^. Direct or indirect application of fungicides can have an adverse impact on soil microbiomes which serve to defend the soil borne fungal pathogens^51^. Soil fungi were particularly sensitive to cantharidin and norcantharidin with a significant decrease in fungal abundance even under the lowest levels of pesticide in the soil (1.25 and 12.5 mg kg^−1^ DW), corresponding to higher concentrations. Previous studies have demonstrated that cantharidin and norcantharidin can significantly inhibit protein serine/threonine phosphatases activity in eukaryotic cells *in vitro* ^17, 23, 52, 53^ and protein serine/threonine phosphatases are widely expressed and relatively conserved among eukaryotes^18^. Therefore, we speculate that the detrimental effect of cantharidin and norcantharidin on soil fungi could be attributed to an inhibitory effect on protein serine/threonine phosphatases, disrupting metabolism and signal transduction^17^. This resembles the AHAS gene, the target of sulfonylurea herbicides, which can be expressed in numerous fungal species. Nicosulfuron, an AHAS-inhibiting compound, has been found to inhibit various fungal strains including soil-borne plant pathogens because of an inhibitory effect on AHAS^12^. Moreover, soil pH also plays a critical role in controlling fungi, keeping bacterial ratios beneficial and maintaining the distribution of functional and taxonomic groups^54^.

Another possible reason of changes in the soil microbial parameters in our study is that these changes not only correlate with the active ingredients, but also involve additives present in commercial formulations. Notwithstanding, there is little information available about the impact of surfactants and adjuvants on soil biota^15, 55^. Pesticide adjuvants such as alcohol ethoxylates and alkylamine ethoxylates are toxic to microeukaryotes and bacteria at low and high concentrations respectively, although some soil microorganisms can use them as C- and N-resources^15, 56^. To our knowledge, this inhibitory effect of cantharidin and norcantharidin has not been previously reported in soil microorganisms. Therefore, the outcomes of this assessment expand our knowledge of the development of environment-friendly pesticides and decision-making (by farmers, advisers and public authorities) concerning the choice of pesticides which is less harmful for soil microorganisms.

Finally, as a potential pesticide of animal origin, cantharidin and its derivatives have attracted wide attention in medical science, but little work has been done in agricultural practice. In recent years, because many insects have developed resistance to various types of pesticides, this new active susbstance characterized by a novel insecticidal mechanism and easy degradation should be studied for agricultural usage. Much work has been done to optimize its structure and, combined with computer simulations, to increase its efficiency in medical and agricultural areas^29, 57^. Soil microbial properties have been shown to be a valuable monitoring tool to assess the impact of pesticide applications on soil health. Particularly, at higher concentrations, non-target effects are more pronounced. Ecologically-relevant methods with increasing pesticide concentrations due to repeated application should be included when estimating their environmental influence and possible non-target effects. Our work provides insights into the potential toxicity assessment of cantharidin and its analogue to non-target organisms, and helps pave the way for their rational utilization in pest control.

## Materials and Methods

### Soil treatment and chemicals

Surface soil samples (top 0–20 cm) were collected from the Teaching and Research Base with perennial *Malus pumila* Mill tree of the Northwest A&F University (34°29′21″N, 108°07′87″E) in Xi’an City on October 2014. After collection, soil samples stored in sealed dark plastic bags were immediately taken to the laboratory. The samples were thoroughly mixed, air-dried at 25 °C for 48 h, and sieved to <4 mm for removing roots, plant debris, rocks and any fauna visible to the naked-eye (>4 mm). The soil is of a Chernozem Calcic character (FAO) with a silt loam texture (clay-silt-sand: 7.2–73.8–19.0%), a pH of 8.53 (1:2.5 w/v in water), a cation exchange capacity of 9.8 cmol/kg, an organic matter content of 3.1%, and with no record of pesticide applications for the last 10 years. A complete randomized block design with three replicates in semi-open plastic bags containing 500 g of soil was employed to investigate the effect of cantharidin (CTD, 3a,7a-dimethylhexahydro-4,7-epoxyisobenzofuran-1,3-dione), norcantharidin (NCTD 7-oxabicyclo [2.2.1] heptane-2,3-dicarboxylic anhydride), and carbendazim [(*RS*)-1-p-chlorophenyl-4,4- dimethyl-3-(1H-1,2,4-triazol-1-ylmethyl) pentane-3-ol] on the soil enzymes. Two new active substances were selected to further explore the mechanisms of the inhibitory effects on fungicidal community. Commercial formulations of applied pesticides were selected because the adjuvants and surfactants existing in various formulations may affect not only soil microorganism populations but also pesticide degradation rates. Our soils were then subjected to the following commercial formulations: a wettable powder containing 50% of carbendazim (Guoguang agrochemical Co., Ltd, Sichuan, China), an emulsifiable concentrate containing 1% cantharidin (from our laboratory) and a microemulsion with 5% norcantharidin (from our laboratory). For each pesticide, three concentrations were used, including a lower recommended field rate (RC) (2 mg kg^−1^ DW soil for carbendazim, 1.25 mg kg^−1^ DW soil for cantharidin and 12.5 mg kg^−1^ DW soil for norcantharidin), a five-fold rate and a highest concentration of 50-fold of the RC. The moisture was monitored gravimetrically and periodically adjusted by adding aseptically distilled water to maintain 60% of the maximum water-holding capacity and natural aeration. Soil microcosm samples were collected at 3, 7, 15 and 35d.

### Determination of different soil microbial parameters

Soil microbial basal respiration was determined following ISO 16072 Norm-2002.

Soil invertase activity was measured by the method of Guan (1986) with slight modification^58^. 2 g fresh soil was placed into a 50 ml Erlenmeyer flask before adding a 15 ml aliquot of 10% (m/m) sucrose solution, 5 ml phosphate buffer (pH 5.5) and 1 ml toluene. After incubation at a constant temperature and humidity incubator for 24 h at 37 ± 1 °C, the sample was filtered through quantitative filter paper. Thereafter, 0.5 ml filtrate was transferred into a 15 ml test tube with 1.5 ml salicylic acid before heating in boiling water for 5 min. This solution was then cooled for 3 min with flowing tap water, and subsequently diluted with deionized water to 10 ml. The absorbance was measured at 508 nm.

Urease was assayed following Tabatabai^59^. Untreated and pesticide-treated soil samples (1 g) were mixed with 20 ml citrate buffer (pH 6.7), 10 ml urea solution (10%, w/v), and 1 ml methylbenzene in covered conical flasks. After incubation for 24 h at 37 ± 1 °C using a shaking table, the mixture was filtered through quantitative filter paper. Then, 5 ml filtrate was transferred into a 50 ml volumetric flask before adding distilled water to about the total volume of 20 ml. Subsequently, 4 ml sodium phenate solution (1.35 mol/L) and 3 ml sodium hypochlorite solution (containing 0.9% active chlorine) were placed into the volumetric flask. After 20 minutes’ rest, this cocktail was measured at 578 nm to detect the NH_4_^+^ released by the urease enzymatic hydrolysis of urea.

For the measurement of alkaline and neutral phosphate activities, 1.0 g of fresh soil was mixed with 0.2 ml toluene, 4 ml of modified universal buffers (MUB) of pH 7.0 and pH 11.0 and 1.0 ml of p-nitrophenyl phosphate (0.025 M). After incubation for 1 h at 37 °C, this mixture was well mixed with 1.0 ml of CaCl_2_ (0.5 M) and 4.0 ml of NaOH (0.5 M) and then filtered through qualitative filter paper. The absorbance was measured at 508 nm.

### Total DNA extraction and fungal community fingerprints

FASTDNA^®^spin kit (MP Biomedicine, USA) for soil was used to extract total microbial DNA directly from each sample according to the manufacturer’s recommendations with FAST beat being replaced by the method of heat-thaw cycles. Recovered DNA extracts were analyzed by 1% agarose gel electrophoresis and stored at −70 °C. Ethidium bromide stain and UV-transillumination showed that the concentration of genomic DNA exceeded 10 ng/μl with an average fragment size above 20 kb in length. The primers used for PCR-DGGE of 18S rRNA gene were FF390 and FR1-GC (with a 40-bp GC clamp at the 5′ end)^60^. For each soil sample, the PCR was performed thrice independently and the amplification products were pooled to minimize the effect of PCR biases. The PCR production was subjected to DGGE (8% (w/v) polyacrylamide gel, 50 v, 18 h) using the Bio-Rad Dcode^TM^ system (Bio-Rad Hercules, CA, USA), with a linear denaturing gradient of 35–50% (100% denaturing gradient: 7 M urea and 40% v/v formamide). At the end, gels were stained using silver nitrate, photographed and digitized with Quantity One^®^ software (Bio-Rad, USA). Selected predominant bands were excised from the DGGE gels and transferred into a PCR tube containing 10 μl of MilliQ sterile water. These tubes were stored overnight at 4 °C and 4 μl of the water was used as the template to amplify DNA in subsequent PCR.

### Monitoring and analysis of cantharidin and norcantharidin

Cantharidin concentration was determined according to Lü *et al*. (2015)^61^. For norcantharidin concentration determination, 10 g soil samples from different incubation times were mixed with 40 ml acetone and extracted three times by a rotary shaker for 30 min. The mixture was then passed through filter paper. We pooled the extracts before dehydrating with anhydrous Na_2_SO_4_; they were then dried completely using a rotary vacuum evaporator. The residues were re-dissolved in 1 ml acetone and quantified by the GC method using an Agilent 4890D (Santa Clara, CA, USA) with a HP-5 capillary column (15 m × 0.53 mm × 1.5 um) filled 101 silylations supporter and flame ionization detector.

### Data analysis

Statistical analyses were performed using SPSS software (SPSS21.0). One-way ANOVA and Fisher’s PLSD post-hoc test were used to compare the differences between controls and samples treated with different concentrations of pesticides, and sampled at a constant incubation time. The significance level was set at 5%. The effect of pesticide type, concentration of pesticides and sampling time on the soil index was determined by three-way ANOVA.

The degradation of cantharidin in soils was fitted to the first-order kinetic model. The rate constant k (day^−1^) was determined using the algorithm equation (1), where C_0_ is the amount of norcantharidin in soil at time zero and C_t_ is the amount of norcantharidin at time t (d). Linear regression was used to calculate the time when the norcantharidin concentration in the soil was reduced by 50% (DT_50_).

1 $${C}_{t}{/C}_{o}={{\rm{e}}}^{-{\rm{kt}}}$$

DGGE gels were analyzed with Quantity One software (Bio-Rad, USA) to calculate the similarity values of the fungal community. Dice coefficient and the unweighted pair-group method using arithmetic averages (UPGMA) were used to construct phylogenic dendrograms on the basis of the band presence/absence and band weighting (band density) analyses. Non-metric multidimensional scaling (nMDS) representations were generated from Bray-Curtis similarity matrices by Past software according to A. Lagomarsino *et al*.^62^. Richness (*S*) values were calculated as the number of DNA bands detected in the respective line of the DGGE profile, while the Shannon-Wiener index (H) and evenness (EH) values were calculated according to the eqs (2) and (3), respectively, where pi is the ratio between the specific band intensity and the total intensity of all bands and S is the total number of bands in each sample.

2 $${{\rm H}}=-\sum {\rm{pi}}({\rm{lnpi}})$$

3 $${{{\rm E}}}_{{\rm{{\rm H}}}}={{\rm H}}/{{{\rm H}}}_{{\rm{\max }}}={{\rm H}}/\,\mathrm{ln}\,{\rm{S}}$$

From the values of the enzyme activities, the treated-soil quality index (T-SQI) proposed by Mijangos *et al*.^27^, was calculated for each incubation time. This index takes into account both (i) the magnitude of the increment of each enzyme activity, as compared to the value for that specific enzyme activity, shown by the reference soil (100%; first ∑ the numerator), and (ii) the maintenance of the evenness among the studied enzyme activities shown by the reference soil (second ∑ of the numerator): equation (4).

4 $${\rm{T}}-{\rm{SQI}}=10\,\mathrm{log}\,{\rm{m}}+\frac{\sum _{{\rm{i}}=1}^{{\rm{n}}}\,\mathrm{log}\,{{\rm{n}}}_{{\rm{i}}}-\sum _{{\rm{i}}=1}^{{\rm{n}}}|\,\mathrm{log}\,{{\rm{n}}}_{{\rm{i}}}-\mathrm{log}\,\overline{{\rm{n}}}|}{{\rm{n}}}$$

Where m is the reference (mean value of enzyme activity in the control, untreated, “reference” soil, set to 100%) and n are the measured values for each enzyme activity as percentages of the reference.

## Electronic supplementary material

Supplementary Information
