# Supplementary material for: Non-target effects on soil microbial parameters of the synthetic pesticide carbendazim with the biopesticides cantharidin and norcantharidin
Source: Sci Rep. 2017 Jul 17;7:5521. doi: 10.1038/s41598-017-05923-8 (PMC5514074; doi:10.1038/s41598-017-05923-8)
Supplement: Supplementary file 1 — Supplementary Information [file 41598_2017_5923_MOESM1_ESM.pdf]

**Non-target effects on soil microbial parameters of the synthetic pesticide carbendazim with the biopesticides cantharidin and norcantharidin**

Hainan Shao and Yalin Zhang*

Key Laboratory of Plant Protection Resources and Pest Management, Ministry of Education, College of Plant Protection, Northwest A&F University, Yangling, Shaanxi 712100, China.

***Corresponding author:** yalinzh@nwsuaf.edu.cn

**Table S1. Relationships between pesticide concentration and microbial parameters at each incubation time.**

| Parameter | Day | CBA | | CTD | | NCTD | |
|---|---|---|---|---|---|---|---|
| | | **Regression** | **$r^2$** | **Regression** | **$r^2$** | **Regression** | **$r^2$** |
| **INV** | 3 | P=-0.07 C+73.965 | 0.0676 | P=-0.29 C+70.65 | 0.263 | P=-0.058•C+98.49 | 0.46 |
| | 7 | P=-0.08•C+61.11 | 0.109 | P=-0.20•C+59.57 | 0.206 | P=-0.020•C+62.02 | 0.268 |
| | 15 | P=-0.11•C+56.43 | 0.562 | P=-0.11•C+56.90 | 0.322 | P=-0.018•C+64.22 | 0.541 |
| | 35 | P=-2.40•C+169.0 | 0.242 | P=-5.36•C+374.78 | 0.471 | P=-0.014•C+63.40 | 0.264 |
| **URE** | 3 | P=-0.0009•C+4.39 | 0.030 | P=-0.0008•C+4.77 | 0.037 | P=-0.0002•C+4.38 | 0.074 |
| | 7 | P=-0.004•C+4.10 | 0.843 | P=-0.0045•C+4.33 | 0.544 | P=-0.0003•C+4.12 | 0.204 |
| | 15 | P=-0.0014•C+3.81 | 0.105 | P=-0.0078•C+4.51 | 0.282 | P=-0.004•C+4.10 | 0.054 |
| | 35 | P=0.0076•C+3.41 | 0.71 | P=-0.0158•C+3.71 | 0.96 | P=-0.0004•C+3.64 | 0.68 |
| **ALK-PHO** | 3 | P=-0.019•C+7.04 | 0.16 | P=-0.016•C+9.36 | 0.49 | P=-0.0025•C+9.56 | 0.74 |
| | 7 | P=0.0014•C+5.83 | 0.014 | P=0.0055•C+5.38 | 0.50 | P=-0.0002•C+5.39 | 0.20 |
| | 15 | P=0.0044•C+6.62 | 0.18 | P=-0.019•C+6.18 | 0.97 | P=0.008•C+5.95 | 0.38 |
| | 35 | P=-0.0012•C+7.52 | 0.21 | P=-0.003•C+7.84 | 0.26 | P=-0.0032•C+7.76 | 0.98 |
| **NEU-PHO** | 3 | P=-0.0016•C+3.05 | 0.60 | P=-0.0047•C+3.15 | 0.99 | P=-0.0003•C+2.86 | 0.15 |
| | 7 | P=-0.0022•C+3.32 | 0.26 | P=-0.0053•C+2.98 | 0.12 | P=-0.0001•C+3.36 | 0.04 |
| | 15 | P=-0.0006•C+3.53 | 0.05 | P=-0.0012•C+3.38 | 0.97 | P=0.0002•C+3.64 | 0.10 |
| | 35 | P=0.0018•C+3.85 | 0.12 | P=-0.0022•C+3.8 | 0.41 | P=-0.0002•C+4.15 | 0.02 |

C: in mg $kg^{-1}$ DW soil; P: for units, see figures.

NEV: invertase activity; URE: urease activity; ALK-PHO: alkaline phosphatase activity; NEU-PHO: neutral phosphatase activity.

**Table S2. Analysis of variance for soil microbial parameters as affected by different factors and corresponding interactions.**

| Factor | INE | URE | PHO-OH | PHO-NEU | *H*'-FUN |
|---|---|---|---|---|---|
| **P** | <0.001 | <0.001 | <0.001 | <0.001 | <0.001 |
| **C** | <0.001 | <0.001 | <0.001 | <0.001 | <0.001 |
| **T** | <0.001 | <0.001 | <0.001 | <0.001 | 0.13 |
| **P ×C** | - | - | - | - | - |
| **P ×T** | 0.001 | 0.27 | 0.006 | <0.001 | 0.27 |
| **C ×T** | <0.001 | 0.15 | <0.001 | <0.001 | <0.001 |
| **P ×C ×T** | - | - | -- | - | - |

P: type of pesticide; C: pesticide concentration; T: incubation time.

INE: invertase activity; URE: urease activity; PHO-OH: phosphatase alkaline; PHO-NEU: phosphatase neutral; *H*'-FUN: Shannon's index of fungal community diversity.
